# Pro-adrenomedullin usefulness in the management of children with community-acquired pneumonia, a preliminar prospective observational study

**DOI:** 10.1186/1756-0500-5-363

**Published:** 2012-07-20

**Authors:** Marta Sardà Sánchez, Joan Calzada Hernández, Susanna Hernández-Bou, Gemma Claret Teruel, Jesús Velasco Rodríguez, Carles Luaces Cubells

**Affiliations:** 1 Emergency Department, Hospital Sant Joan de Déu, Universitat de Barcelona, Passeig Sant Joan de Déu 2, 08950, Esplugues de Llobregat, Barcelona, Spain; 2 Laboratory Department, Hospital Sant Joan de Déu, Universitat de Barcelona, Passeig Sant Joan de Déu 2, 08950, Esplugues de Llobregat, Barcelona, Spain

**Keywords:** Pro-adrenomedullin, Pneumonia, Children

## Abstract

**Background:**

In adult population with community acquired pneumonia high levels of pro-adrenomedullin (pro-ADM) have been shown to be predictors of worse prognosis. The role of this biomarker in pediatric patients had not been analyzed to date. The objective of this study is to know the levels of pro-ADM in children with community acquired pneumonia (CAP) and analyze the relation between these levels and the patients’ prognosis.

**Findings:**

Prospective observational study including patients attended in the emergency service (January to October 2009) admitted to hospital with CAP and no complications at admission. The values for pro-ADM were analyzed in relation to: need for oxygen therapy, duration of oxygen therapy, fever and antibiotic therapy, complications, admission to the intensive care unit, and length of hospital stay. Fifty patients were included. Ten presented complications (7 pleural effusion). The median level of pro-ADM was 1.0065 nmol/L (range 0.3715 to 7.2840 nmol/L). The patients presenting complications had higher levels of pro-ADM (2.3190 vs. 1.1758 nmol/L, p = 0.013). Specifically, the presence of pleural effusion was associated with higher levels of pro-ADM (2.9440 vs. 1.1373 nmol/L, p < 0.001).

**Conclusions:**

In our sample of patients admitted to hospital with CAP, pro-ADM levels are related to the development of complications during hospitalization.

## Findings

### Background

In recent years several clinical and analytical scales have been developed to evaluate severity and prognosis of adult patients with community-acquired pneumonia (CAP), such as *Pneumonia Severity Index* (PSI) [1], CURB-65 and CRB-65 [2]. Furthermore, many studies have examined the usefulness of diverse biomarkers in the diagnostic, etiological, and prognostic classification of these patients [3-5]. One of the biomarkers evaluated is pro-adrenomedullin (pro-ADM) [6,7]. This is a peptide originally isolated in human pheochromocytoma in 1993 with a powerful vasodilating and bactericide effect. High levels of pro-ADM have been shown to be predictors of seriousness and mortality in various pathologies in adult population [6-9]. Specifically, in CAP, two recent studies have shown that patients with high levels of pro-ADM present a worse prognosis [6,7]. The role of pro-ADM in a pediatric population had not been analyzed to date.

Objectives of our study were to determine the levels of pro-ADM in pediatric patients suffering CAP, and to analyze the relation between these levels and the patients’ prognosis.

### Study population

The study was carried out in a tertiary-care children’s hospital with 275 beds and an average referral population of 1,200,000 children under the age of 18 years, and 105,485 patients attended in the Emergency Service (ES) every year.

This is a prospective observational study including patients under 18 years of age attended between January and October 2009 in the ES and hospitalized with CAP as the main diagnosis, and blood work carried out at admission. CAP was defined as acute pulmonary inflammation involving the aerial and interstitial space of infectious origin, according to the classical combination of clinical and radiological findings (code 486 in the diagnosis coding of the Spanish Emergency Pediatrics Society). Chest radiographs were screened by the physician in charge and reviewed by a senior radiologist. Patients were excluded if they had an immunocompromising or chronical medical condition predisposing them to severe pneumonia (chronic cardiopulmonary disease, primary or acquired immunodeficiency, neuromuscular disease or cerebral palsy). Those patients with complicated CAP at the time of admission and those receiving oral or parenteral antibiotic treatment for a previous diagnosis of CAP were also excluded.

For the purposes of the present study we considered complicated CAP when one of the following was present: pleural effusion, cavitation, lung abscess or necrosis, air escape, massive atelectasis or signs of systemic infection (sepsis).

Admission criteria in our center for patients with CAP are age under three months, age under one year with moderate-to-severe symptoms, any age with severe clinical state, underlying illness susceptible to decompensation, involvement of more than one lobe or extensive unilobar involvement, pleural effusion, cavitary pneumonia or lung abscess, pneumonia that failed to respond to correct antibiotic treatment begun in the previous 48–72 hours, oral intolerance or suspected therapeutic non-compliance. The physician in charge decides on the need for blood work or for initiating endovenous antibiotic treatment. When blood work was carried out, determination of pro-ADM levels were also performed. Pro-ADM levels were known after the discharge of the patients.

To know whether pro-ADM levels were correlated with prognosis, its levels were measured in relation to the following parameters: need for oxygen therapy, duration of fever, oxygen and parenteral antibiotic therapy during hospitalization, length of hospital stay, admission to Intensive Care Unit (ICU), and development of complications. In our hospital oxygen therapy is indicated when percutaneous oxygen saturation is under 95% or when significant respiratory distress is present. In addition, pro-ADM levels were also compared according to the site of pneumonia (unilobar versus multilobar) at admission.

The study was approved by the Local Human Investigation Ethics Committee. Verbal informed consent was obtained from all parents or legal tutors of the patients before inclusion in the study.

### Processing of the sample

EDTA was added to the blood sample in order to measure pro-ADM. For its measurement in blood serum, TRACE (*time-resolved amplified cryptase emission*) technology was used by Kryptor Compact pro-ADM ® (Brahms, Hennigsdorf, Germany). This system has an optimized functional sensitivity (inter-assay precision: 20%) of 0.25 nmol/L.

### Statistical analysis

Data analysis was conducted using SPSS 17.0 software. The study population was described using frequencies and percentages for categorical variables and means, standard deviations, medians, ranges and interquartile ranges (IQR) for continuous variables. Statistical comparisons were made using a chi-square test or Fisher exact test for categorical data, and a student’s t-test or Mann–Whitney U test for continuous data. Dependence between continuous variables was determined with Pearson’s correlation. P values less than 0.05 were considered significant.

## Results and discussion

During the study period 2066 patients were diagnosed with CAP in the ES. A total of 298 were admitted to hospital (14.4%); 187 of them were excluded for presenting any of the mentioned exclusion criteria. Of the 111 remaining patients, 61 were excluded because there was not blood work done at admission (19 cases) or because there was no pro-ADM in the blood sample taken at admission (42 cases). The final sample was composed of 50 patients. There were no statistically significant differences between the clinical characteristics of these last two groups at the evaluation in the ES (Table 1).

**Table 1:** Clinical characteristics at admission of the 50 patients included in the study and the 61 patients excluded because of the lack of pro-ADM levels

	***Included patients (n = 50)***	***Excluded patients (n = 61)***	***p***
Female sex, n (%)	26 (52)	28 (45.9)	*0.327*
Age	4.5 years (1.9 months to 13.6 years)	2.5 years (23 days to 12.9 years)	*0.74*
Maximum temperature, °C	39.5 (38–40.7)	39 (37–41.2)	*0.41*
Duration of fever, days	3 (0.2 to 7)	3 (0–21)	*0.838*
Respiratory distress, n (%)	26 (52)	42 (68.8)	*0.053*
Percutaneous oxygen saturation < 95%, n (%)	16 (32)	25 (40.9)	*0.159*
Chest radiograph			
- Unilobar infiltrate, n (%)	36 (72)	39 (63.9)	0.512
- Multilobar infiltrate, n (%)	10 (20)	13 (21.3)	
- Interstitial infiltrate, n (%)	4 (8)	9 (14.7)	

The values are displayed as medians and ranges, unless otherwise indicated.

Of the 50 patients included in the study, 18 had received at least 2 doses heptavalent pneumococcal conjugate vaccine. Thirty (60%) received oxygen therapy during admission, with a median duration of two days (range 0.5 to 11). A total of 42 patients presented fever during admission (84%), with a median duration of one day (range 0.5 to 10). All patients received antibiotics for a median time of 4 days (range 2 to 24). During the hospital stay 10 patients (20%) presented complications: pleural effusion (7 cases), pulmonary abscess (two cases), and massive atelectasis (one case). Six patients with pleural effusion required pleural drainage, in two cases with prior thoracoscopy. Five children (10%) required admission to ICU, all cases with non-invasive ventilation and two with mechanical ventilation. All 5 patients presented a good evolution. Median length of stay was 5 days (range 2 to 25).

**Figure 1:**
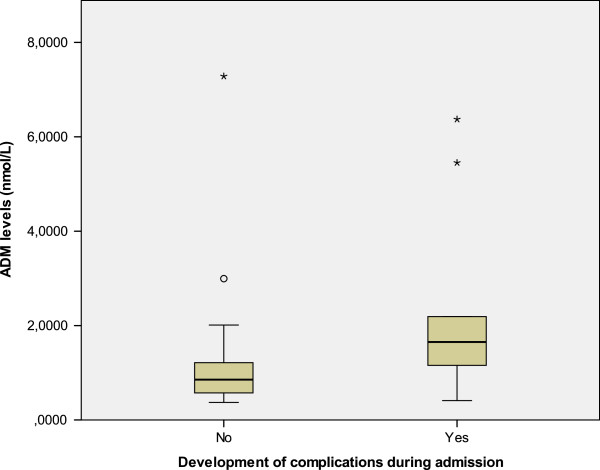
Pro-adrenomedullin levels in the 50 patients according to the development of complications during admission.

The median level of pro-ADM was 1.0065 nmol/L (range 0.3715 to 7.2840, IQR 0.7598 to 1.3588). The patients with complicated pneumonia had higher levels of pro-ADM than those who did not develop complications (2.3190 *vs.* 1.1758 nmol/L, p = 0.013), as we show in Figure 1*.* Concretely, the patients with pleural effusion had a median level of pro-ADM of 2.9440 *vs.* 1.1373 nmol/L for the patients not presenting this complication (p < 0.001). C-reactive protein (CRP) levels not differ between patients with uncomplicated and uncomplicated CAP (206 *vs.* 254.5 mg/L, p = 0.558). No statistically significant relation was found between pro-ADM levels and the other variables analyzed: need for oxygen therapy (1.5 *vs.* 1.3, p = 0.8), duration of fever during hospital stay (Pearson correlation index 0.2; p = 0.2), duration of oxygen therapy (Pearson correlation index 0.01; p = 0.9), length of hospital stay (Pearson correlation index 0.2; p = 0.2), duration of parenteral antibiotic therapy (Pearson correlation index 0.2; p = 0.1), admission to ICU (1.4 *vs.* 0.9, p = 0.5) and unilobar or multilobar CAP at admission (1.1 *vs.* 0.9, p = 0.585).

To our knowledge this is the first study that evaluates the levels of pro-ADM in pediatric patients with CAP. Our purpose was analyzing the usefulness of this parameter as a predictor of patient evolution.

Two main mechanisms have been described as responsible for the increase of circulating pro-ADM in infections, including CAP, which can be regarded as a precursor of sepsis. Firstly, as a member of the calcitonin gene family, ADM is widely expressed and extensively synthesized during severe infections. Bacterial endotoxins and proinflammatory cytokines up-regulate ADM gene expression in many tissues. In addition, a decreased clearance by the kidneys may be responsible in part for the increased pro-ADM levels in infections [10].

The median level of pro-ADM found in our study was similar to those found by Christ-Crain *et al*. [6] and Huang DT *et al*. [7] in adult population with CAP: 1.1 nmol/L (IQR 0.7-1.9) and 1.0 nmol/L (IQR 0.7-1.5) respectively.

In our study higher levels of pro-ADM at the time of diagnosis correlated with further development of complications during hospital stay, especially pleural effusion. However, there was an overlapping of pro-ADM’s levels between the patients presenting complications and the rest. This issue could be probably addressed in a larger study. None of the published studies evaluating pro-ADM levels in adult population with CAP analyzed specifically the relation between pro-ADM levels and development of pleural effusion during admission. Christ-Crain *et al*. [6] included 302 patients and observed that pro-ADM levels correlated with increasing severity of illness according to the PSI score and death. They proposed a cut-off point of 1.8 nmol/L (80% sensitivity, 72% specificity). A later multicenter study by Huang DT *et al*. [7] including 1653 patients found higher levels of pro-ADM in patients stratified into higher risk groups according to the PSI and the CURB-65 scores. They also found that higher levels of pro-ADM at admission were associated with an increased need for intensive care, vasopressors, and increased mortality. The cut-off proposed by the authors was 1.3 nmol/L (68% sensitivity, 73% specificity). Recently, Albrich et al. [11] in a multicenter study including 1359 adult patients with lower respiratory tract infection proposed a new score combining CURB65 risk classes with pro-ADM levels to predict adverse events and mortality. In other recent study, Bello *et al.*[12] confirmed pro-ADM high short- and long-term prognostic accuracy, and that this biomarker increased the accuracy of clinical scores. In the mentioned manuscripts the prognostic value of MR-proADM was not modified by different possible CAP aetiologies. In our study we could not address this issue due to lack of information about CAP aetiology. Suberviola et al. [13] included 49 patients with severe sepsis or septic shock due to CAP. Pro-ADM levels correlated with death and increasing severity of illness, levels rose as PSI class advanced.

The present study has several limitations: 1) Patients discharged were not included because, according to our protocol, these patients usually don’t need blood work; for that reason pro-ADM levels obtained are not representative of general pediatric population with CAP; 2) Strict exclusion criteria were applied in order to insure a homogeneous sample; again, results obtained can’t be applied to all pediatric patients hospitalized with CAP; 3) Furthermore we excluded a relevant number of patients in whom pro-ADM determination was not obtained. This fact could have introduced a bias into the pro-ADM values obtained, because patients without blood sample had probably a mildness disease. Nevertheless, comparison between two groups showed no statistically significant differences in clinical characteristics at admission. In addition, values obtained in our sample were similar to those obtained in the studies of CAP in adults that included patients admitted to hospital and those discharged [6,7]. As a consequence of these limitations, the final sample size was small and we were not able to establish prognosis cut-off values or to analyze subgroups of patients.

## Conclusions

In conclusion, in our sample a higher level of pro-ADM at admission was related to a greater likelihood of complications during the hospital stay and therefore it could be helpful in the management of patients with CAP in the ES. Nevertheless, future studies with larger samples will be needed to confirm our results and to analyze the association of pro-ADM levels with other variables of prognostic significance.

## Abbreviations

pro-ADM, Pro-Adrenomedullin; CAP, Community Acquired Pneumonia; PSI, Pneumonia Severity Index; ES, Emergency Service; ICU, Intensive Care Unit; TRACE, Time-Resolved Amplified Cryptase Emission; IQR, Interquartile Ranges; CRP, C-reactive protein.

## Competing interests

The authors declare that they have no competing interests.

## Authors’ contributions

MS and JC collected the data, analyzed them, and drafted the article. SH and GC conceived the study and participated in its design, coordination, data analysis and helped to draft and revising the manuscript. JV carried out the laboratory tests to determine pro-ADM values. CL revised critically the manuscript, participating in the final redaction. All authors read and approved the final manuscript.

## Funding

This work was supported in part by the Thermo Fisher Scientific (Hennigsdorf, Germany) that provided the Pro-ADM ® kit for the study. The authors declare no other sources of funding.
